# Environmental surveillance for *Salmonella* Typhi to detect the typhoid burden in Yogyakarta, Indonesia

**DOI:** 10.1016/j.ijheh.2025.114572

**Published:** 2025-05

**Authors:** Vicka Oktaria, Indah Kartika Murni, Amanda Handley, Celeste M. Donato, Titik Nuryastuti, Endah Supriyati, David T. McCarthy, Emma Watts, Rizka Dinari, Hendri Marinda Sari, Jarir At Thobari, Ida Safitri Laksono, Julie E. Bines

**Affiliations:** a Department of Biostatistics, Epidemiology, and Population Health, Faculty of Medicine, Public Health, and Nursing, Universitas Gadjah Mada, Yogyakarta, Indonesia; b Center for Child Health - Pediatric Research Office, Faculty of Medicine, Public Health, and Nursing, Universitas Gadjah Mada, Yogyakarta, Indonesia; c Child Health Department, Faculty of Medicine, Public Health, and Nursing, Universitas Gadjah Mada, Yogyakarta, Indonesia; d Medicines Development for Global Health, Southbank, Victoria, Australia; e The Peter Doherty Institute for Infection and Immunity, The University of Melbourne, Victoria, Australia; f Enteric Diseases Group, Murdoch Children's Research Institute, Parkville, Victoria, Australia; g Department of Microbiology, Faculty of Medicine, Public Health, and Nursing, Universitas Gadjah Mada, Yogyakarta, Indonesia; h Center for Tropical Medicine, Faculty of Medicine, Public Health, and Nursing, Universitas Gadjah Mada, Yogyakarta, Indonesia; i Environmental and Public Health Microbiology Lab (EPHM Lab), Department of Civil Engineering, Monash University, Clayton, Victoria, Australia; j School of Civil and Environmental Engineering, Faculty of Engineering, Queensland University of Technology, Queensland, Australia; k Department of Paediatrics, The University of Melbourne, Parkville, Victoria, Australia; l Department of Gastroenterology and Clinical Nutrition, Royal Children's Hospital Melbourne, Victoria, Australia

**Keywords:** Wastewater-based epidemiology, Surveillance, Typhoid fever, *Salmonella* Typhi, Indonesia

## Abstract

**Background:**

In low and middle-income countries (LMICs), understanding the burden of typhoid disease has been challenging as clinical surveillance based on blood culture data alone often poorly represents the community burden. Underreported cases, unclear case definitions, the presence of a chronic carrier state and emerging antimicrobial resistance necessitate alternative approaches to assess disease prevalence and target public health interventions, such as vaccine introduction. This study aimed to assess the feasibility of wastewater and environmental surveillance (WES) in measuring the prevalence of typhoid infection in Indonesia.

**Methods:**

Between October 11, 2022, and August 31, 2023, WES was conducted in 18 locations across 3 districts in Yogyakarta province, Indonesia. Samples were collected fortnightly from wastewater treatment plants (WWTPs), manholes, a river, and public spaces, using grab and passive sampling methods. *Salmonella* Typhi (*S.* Typhi) detection was conducted using quantitative PCR for *S.* Typhi genes (*ttr, tviB,* and *staG* – all positive).

**Results:**

Of the 406 samples collected, 13 % (51/406) tested positive for *S.* Typhi, with monthly positivity rates ranging from 2 % (1/51) in March 2023 to 47 % (16/34) in October 2022. Mean concentrations (in log10) in *ttr*, *tviB*, and *staG* in grab samples were 0.67 (SD ± 0.99), 0.23 (SD ± 1.14), and −0.11 (SD ± 1.05). The highest detection rates were observed in samples from the river compared to central WWTPs (OR 12.68; 95 % CI 2.03–79.20, *P* = 0.007). No correlation was observed between rainfall and *S.* Typhi gene detection (*P* > 0.05 for all genes).

**Conclusion:**

WES is feasible in Indonesia and can be used to monitor typhoid disease burden in an endemic region. High positivity rates from the river and septic tanks in traditional markets support a broad approach to sampling in LMICs where formal wastewater management systems may not accurately represent community disease prevalence due to its low population coverage. WES can be a valuable tool to inform public health responses, including vaccine introduction.

## 1 Introduction

Globally, typhoidal Salmonellae (*Salmonella enterica* serovar Typhi and Paratyphi) infection is estimated to cause 11–21 million cases of Typhoid Fever and is associated with 128,000–161,000 deaths per year (Ajibola et al., 2018; World Health Organization, 2018). Although typhoid disease can affect all ages, the greatest burden is in infants, children, and adolescents (Stanaway et al., 2019). Despite this high global burden, there is limited country-level data on typhoid burden in low- and middle-income countries (LMICs), including Indonesia (Antillón et al., 2017). This data is pivotal to informing countries to prioritise public health interventions, including the introduction of typhoid vaccines, to address the mortality and healthcare burden associated with typhoid disease.

Typhoid disease is caused by infection with *Salmonella enterica* serovars Typhi (*S.* Typhi) and Paratyphi (*S*. Paratyphi), which is primarily transmitted through faecally contaminated water and food (Parry et al., 2002). After ingestion, the bacteria invade the small intestinal mucosa and can enter the bloodstream and lymphatic system and spread to the liver, spleen, and bone marrow. Acute infection typically presents clinically as acute typhoid fever, with fatigue, headache, nausea, rash, and either constipation or diarrhea commonly reported. Colonisation of the gallbladder can occur, resulting in chronic *Salmonella* carriage. Patients with acute and chronic infection can transmit *Salmonella* via infected faeces (Parry et al., 2002). This presents a significant risk in LIMCs with limited or lacking effective wastewater management systems.

A key challenge in accurately defining the clinical burden of typhoid disease in LIMCs reflects the current lack of precision in confirming the diagnosis of typhoid infection in many LMICs. The diagnosis is confirmed by the detection of *S.* Typhi in blood culture. *S.* Typhi may also be detected in stool and urine. However, blood cultures are not routinely collected from patients with suspected typhoid fever in many LMICs, including Indonesia, due to cost and limitations in laboratory facilities. Many patients take antibiotics for empirical treatment of fever prior to presentation to a Healthcare facility and/or collection of blood culture, so a negative blood culture likely underestimates the burden of typhoid infection (Ajibola et al., 2018; Antillón et al., 2017; Mina et al., 2023; Obaro et al., 2017). The incidence of antimicrobial resistance is also a growing concern and has been responsible for large outbreaks, which was a key factor that resulted in the introduction of the Typhoid Conjugate Vaccine into the National Immunisation Program in Pakistan (Matrajt et al., 2020). In the absence of a blood culture, in many LMICs, the diagnosis of typhoid fever is based on clinical symptoms and/or a positive blood spot antigen test (such as the Widal test or Typhidot). Although this approach is pragmatic in a low-cost healthcare setting, it lacks sensitivity and specificity.

As typhoid disease can occur both in an acute and chronic clinical phase, with both potentially infective via faecal contamination, the challenge in understanding the population burden of typhoid disease extends beyond the healthcare setting into the community. Given that typhoid can rapidly transition from an active outbreak to an endemic (Matrajt et al., 2020), relying solely on clinical surveillance may be insufficient for early detection of evolving disease dynamics. As an enteric pathogen that is shed in faeces, wastewater and environmental surveillance (WES) may provide an important insight into the population burden and patterns of typhoid disease in LMICs and may serve as a viable and low-cost alternative to population-based studies (Uzzell et al., 2023).

Typhoid infection is considered endemic in Indonesia as stated by the Indonesian Ministry of Health in 2006 (Ministry of Health of the Republic of Indonesia, 2006). The incidence of typhoid disease in the Southeast Asia region has generally declined over the past two decades. A similar trend was shown in Indonesia, as reported by Global Burden of Diseases (GBD), with estimated cases of typhoid of 342.52 cases per 100,000 population in 2007 to 168.73 cases per 100,000 population in 2021 (Institute for Health Metrics and Evaluation (IHME), 2021). These cases definition utilised ICD 9 and 10 codes which are primarily based on clinical manifestation. However, it is unclear if this reflects a true decrease in disease burden. Hence, Indonesia is actively considering the introduction of a locally manufactured Typhoid Conjugate Vaccine (BioFarma Indonesia) and updated data on typhoid disease burden is critical to inform this decision.

This study aims to assess the feasibility of WES to estimate the population prevalence of *S.* Typhi infection in Indonesia. This study is based in the Yogyakarta region that successfully introduced WES which aimed to assist with the public health response during the recent COVID-19 pandemic. WES sites, sample collection protocols, and laboratory methods implemented for SARS-CoV-2 surveillance were adapted for the detection and analysis of *S*. Typhi. Only 6 % of the population (25,294 households) in Yogyakarta included in this study are connected to the central wastewater treatment plant managed by the provincial government. The remainder of the population's wastewater is managed by community wastewater treatment plants, septic systems or directly drain into rivers and waterways. To ensure a broad representation of the typhoid prevalence within this population, samples were collected and analysed from formal wastewater management systems as well as informal systems, rivers, and waterways.

## 2 Material and methods

### 2.1 Study design, sites, and sample collection

**Fig. 1:**
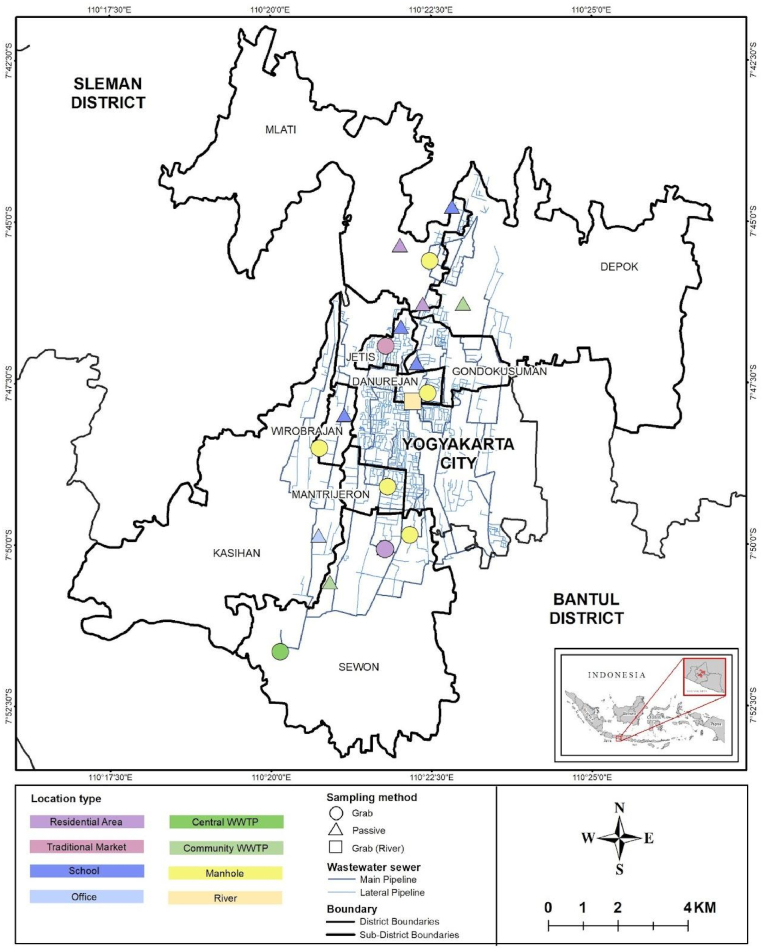
Map of 18 sampling sites in three districts within the Yogyakarta Province. Different colours represent different types of sampling sources. Triangle refers to collection using passive samplers, circle refers to collection using grab sampling method, and rectangle refers to grab sampling method in the river. The map was generated utilising ArcGIS Pro 3.4. (For interpretation of the references to colour in this figure legend, the reader is referred to the Web version of this article.)

Wastewater samples were collected from three regions of Yogyakarta province - Yogyakarta (urban), Sleman (semi-urban), and Bantul (rural), between October 11, 2022 and August 31, 2023. Site selection was based on the wastewater treatment plant (WWTP) coverage, population density, areas where people congregate, and data on the location of suspected typhoid cases provided by local district offices during 2022. A total of 18 wastewater sites were selected across sub-districts in Bantul (Kasihan and Sewon), Yogyakarta City (Jetis, Danurejan, Mantrijeron, Wirobrajan, and Gondokusuman), and Sleman (Depok and Mlati) (Fig. 1, Table A.1). Wastewater samples were collected from the central WWTP (inlet), community WWTPs (inlet), from sites where people commonly congregate (traditional markets, apartments/flats, dormitories, schools, and factories) and from the river where it passes through densely populated and informal settlements (Fig. 1). Samples were collected from manholes receiving residential flow before entering the main pipes.

Samples were collected fortnightly from designated sites identified through the Global Positioning System (GPS) using either a grab or passive sampling methods (as summarised in Table 1) (Habtewold et al., 2022; Murni et al., 2022; Schang et al., 2021; West et al., 2023; Wilson et al., 2022). These methods of sampling are more common in LMICs due to their adaptiveness and practicality compared to composite sampling that is frequently used in high income countries (HICs) (Keshaviah et al., 2023). The utilisation of automatic samplers to collect composite sampling is often impractical in small catchment settings due to several challenges, including high costs for procurement and maintenance, the requirement for skilled personnel to install and operate the machine, limited space for installation (e.g., small manhole spaces or septic tank inlet pipes), restricted access to reach sampling locations, operational difficulties in sub-zero temperatures, and insufficient wastewater flow rates (Wilson et al., 2022).

**Table 1:** Summary of samples by sampling sources.

Sampling source	Number of sites (N = 18)		Number of samples (N = 406)		Collection batch
	n	%	n	%	
Grab method (N = 9)					
Manhole	5	27	120	30	1 to 24
Central WWTP	1	6	24	6	1 to 24
Residential area	1	6	24	6	1 to 24
River	1	6	24	6	1 to 24
Traditional market	1	6	24	6	1 to 24
Passive sampler method (N = 9)					
Community WWTP	2	11	24	6	1 to 24
Residential area	2	12	48	12	1 to 24
School	4	22	70	17	3 sites: batch 1 to 24 1 site: batch 1 to 7 1 site: batch 10 to 24
Office	1	6	24	6	1 to 24

Grab samples of 500 mL of wastewater were collected from manholes, the WWTP, and septic tanks and 2,000 mL was collected from the river using a sterile scoop or a bottle. Passive sampling used a torpedo-style, 3D-printed, plastic hollow container containing one q-tip, medical gauze swab, and two laboratory-grade electronegative membranes (Schang et al., 2021). These samplers were deployed and left for 24 h before collection (Habtewold et al., 2022; Schang et al., 2021). Each selected sampling location was collected using one sampling method. The grab samples and passive samplers were collected at the same time between 7 a.m. and 10 a.m. Samples were placed immediately in a cool box and transferred on ice at 2–8 °C within 4 h of collection to the Microbiology laboratory at the Universitas Gadjah Mada, Yogyakarta.

Personal protective equipment (PPE) was used throughout the sample collection process, and strict infection control measures were implemented to minimise the potential risks to personnel during collection and reduce the likelihood of sample contamination (CDC, 2022; US Environmental Protection Agency, 2013). Sample characteristics were measured using visual inspection (colour and volume) and environmental factors (rainfall [Center for Hydrometeorology and Remote Sensing (CHRS), website https://chrsdata.eng.uci.edu/], air temperature [https://goweatherforecast.com], and water depth) was recorded.

### 2.2 Laboratory method

#### 2.2.1 Sample processing and filtration

Upon arrival at the laboratory at the Universitas Gadjah Mada, Yogyakarta, the bottles containing the grab samples and torpedo passive samplers were stored at 2–8 °C for a maximum of 2 h until processing. For the filtration process, we used one membrane filter of 47 mm diameter, 0.45 μm pore size cellulose nitrate high flow electronegative membrane (Sartorius, Germany), following the previous study (Murni et al., 2022). The torpedo passive samplers were opened, the filter membranes and q-tips were collected. The processed filter membranes (from grab samples) and the filter membranes and q-tips from the passive samplers were stored at −80 °C until DNA extraction.

#### 2.2.2 DNA extraction

The processed (from grab samples) and passive filter membranes were extracted using the QIAamp Powerfecal Pro DNA (QIAGEN, Germany) following the manufacturer's instructions, except for using Hard & Fibrous Tissue Mix 0.7 mm Garnet Beads (Omni International, US) instead of the supplied beads. To monitor the DNA extraction process and PCR inhibition, the qPCR DNA Extraction and Inhibition Control CY5-QXL670 (Eurogentec, Belgium) was used as the internal control. The extracted DNA was either processed directly or stored at −80 °C until the Quantitative Polymerase Chain Reaction (qPCR) process was conducted.

#### 2.2.3 Quantitative polymerase chain reaction (qPCR)

Multiplex qPCR was performed with primers and probes targeting the genes *ttr, tviB*, and *staG* of *S.* Typhi; with all three gene targets required for a sample to be deemed positive (Abraham et al., 2022). Additionally, qPCR was performed to detect HF183, the marker genes from human-restricted *Bacteroides*, as an indicator for human faecal biomass in the samples. Primer and probe sequences used in this study were as previously described (Abraham et al., 2022; Ahmed et al., 2019) and are detailed in Table A.2 and A.3. Cycling conditions for all qPCR reactions were as follows: 50 °C for 2 min, then 95 °C for 15 min, followed by 40 cycles of 95 °C for 30 s, 60 °C for 30 s, and 72 °C for 30 s. A standard curve for each target was produced by running a qPCR on a dilution series of gBlocks DNA fragments (Integrated DNA Technologies/IDT, USA). To increase the sensitivity of *S.* Typhi identification, samples that tested positive for *ttr* and either *staG* or *tviB* alone were retested in a singleplex qPCR for the negative target (Fig.A.1). Cut-off cycle threshold (Ct) values for *ttr, staG,* and *tviB* were 38, 39, and 39, respectively, by evaluating each target's serial dilution of the gBlocks DNA fragments (IDT, USA) (Abraham et al., 2022).

After each PCR, we analysed the normalised Ct values of each gene target in every sample. We determined positivity by comparing the Ct values against the cut-off values determined during the standards run. If all gene targets exhibited Ct values higher than the cut-off, we inspected the Ct values for the target HF183 and the spike-in control. A negative or lower-than-expected result for the qPCR DNA Extraction and Inhibition Control CY5-QXL670 (Eurogentec, Belgium) that spiked into samples in the DNA extraction process could indicate potential issues with PCR inhibition or DNA extraction. Subsequently, if the HF183 target was negative and the spike-in control appeared satisfactory, it might suggest the absence or insufficient amount of faecal contamination at the sample site. The limit of detection (LOD) for the qPCR assay was determined by analyzing ten replicates for each dilution of the gBlocks gene fragment DNA standards (IDT, USA). The LOD was defined as the lowest number of copies of the *ttr*, *tviB*, and *staG* genes detectable in at least 80 % of the replicates, which was determined to be five copies per reaction. To ensure accurate quantification, reflecting the exponential amplification capacity of the assay under optimal conditions, qPCR efficiency was evaluated. It was determined using a standard curve constructed by plotting the Ct values against the logarithm of known DNA concentrations from a serial dilution series. Acceptable efficiency ranges between 90 and 110 % (−3.6 ≥ slope ≥ −3.1), with values outside this range indicating technical issues or suboptimal reaction conditions that must be addressed.

### 2.3 Statistical analysis

All statistical analyses were performed using RStudio version 3.6.3. Descriptive data analysis was presented, and then the proportion of positive samples was calculated based on month, location type, and sub-district. The numerator was the count of positive samples per month/location type, sub-district, and compared to the denominator, i.e., total collected samples per month/location type/sub-district. Gene concentration was reported in the log form of the copies per sample type (copies per mL for grab samples and copies per passive for torpedo passive samplers). The formulae for calculating genome copies per sample type is:

$$\frac{gene\;copies\;per\;reaction/\;volume\;of\;DNA\;\left(\mu L\right)}{volume\;of\;elution\;\left(\mu L\right)/\;volume\;of\;sample\;processed\;\left(mL/passive\right)}$$

We utilised Generalised Linear Mixed Model (GLMM), that consider the week of sampling as a random effect, to assess: 1) the correlation between mean rainfall (mm), as a dependent variable, and the overall proportion of positive samples; 2) the correlation between mean rainfall (mm), as dependent variable, and each gene (*ttr*, *tviB*, *staG*, HF183) only for positive samples as the independent variables; and 3) bivariate analysis between proportion of positive samples (dependent variable) and location type for sampling (independent variable). A comparison between the proportion of positive samples and rainfall was conducted due to the possibility of having lower bacterial concentration in wastewater during rainy weather, as reported in a previous study that assessed the concentration load of *E. coli* (Lucas et al., 2014), and the recovery efficiencies of poliovirus in river (compared to dry weather) (Hata et al., 2014). In addition, maps were generated using ArcGIS Pro 3.4.

### 2.4 Ethics

Ethical approval was obtained from the Medical and Health Research Ethics Committee (MHREC), Faculty of Medicine, Public Health, and Nursing, Universitas Gadjah Mada DR. Sardjito General Hospital, Indonesia (reference number: KE/FK/0426/EC/2021, KE/FK/0514/EC/2022).

## 4 Results

A total of 406 samples were collected in 24 fortnightly batches from 18 sampling sites. This included 216 (53.2 %) grab samples and 190 (46.8 %) samples collected using the passive sampler. One site (school) was discontinued after the 7th sample collection due to the permanent closure of the septic tank cover and replaced with a site with similar characteristics, starting from the 10th sampling batch. As a result, 17 sampling sites were included in each batch, except for the 8th and 9th batches, which had only 16 sites during the transition period. The other 16 sites had 24 samples each. Almost a third of all samples (30 %, 120/406) were collected from the five manholes across the three study districts. Samples collected from WWTPs represented 72 of 406 samples, with 24 (6 %) from central WWTPs and 48 (12 %) from two community WWTPs. Samples were collected from three residential areas (18 %, 72/406), four schools (17 %, 70/406), a river (6 %, 24/406), a traditional market (6 %, 24/406), and an office (6 %, 24/406, Table 1).

**Fig. 2:**
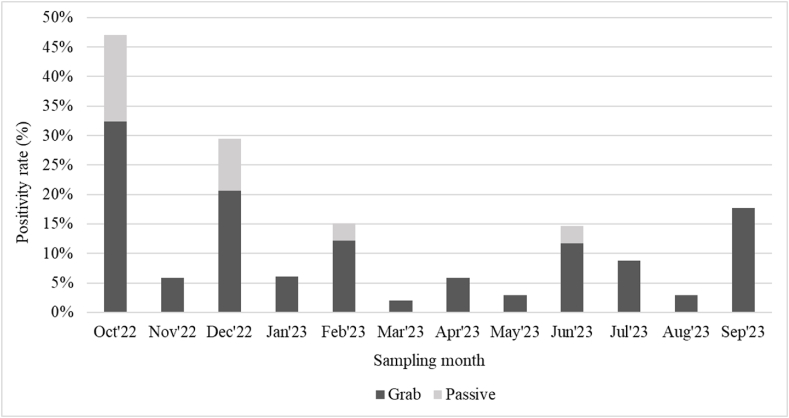
Positivity rate by sampling month (N = 406).

**Fig. 3:**
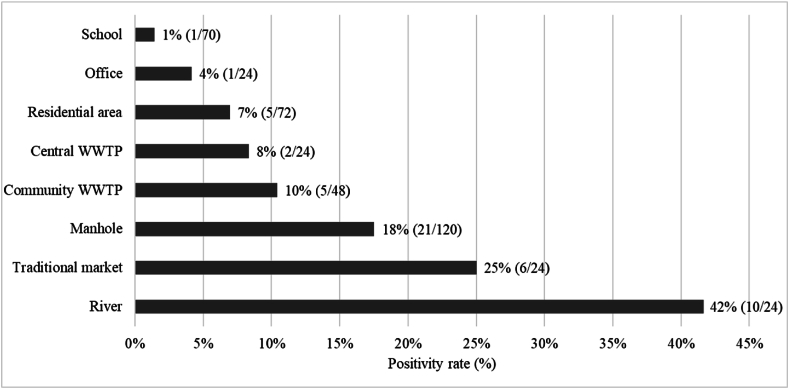
The proportion of positive samples by sampling sources (N = 406).

Over the study period, a total of 51 of 406 samples (13 %) tested positive for *S.* Typhi with all three genes (*ttr, staG,* and *tviB)* positive. Of these samples, 95.32 % (387/406) of all samples collected and 100 % (51/51) of the *S.* Typhi positive samples showed positivity in HF183, confirming that the samples contained human fecal biomass (Table A.4). We observed a seasonal variation in positivity rates from 2 % (1/51) in March 2023 to 47 % (16/34) in October 2022 (Fig. 2). A higher proportion of positive samples was detected from October 2022 (47 %, 16/34) to February 2023 (15 %, 5/33), although positive detections from river samples remained the highest and were consistent throughout the sampling period (10 out of 24 batches, 42 %, from the beginning to the end of sample collection, Fig. 3). There was no significant correlation between rainfall and the positive detection rate (P > 0.05, Table. A.5).

**Fig. 4:**
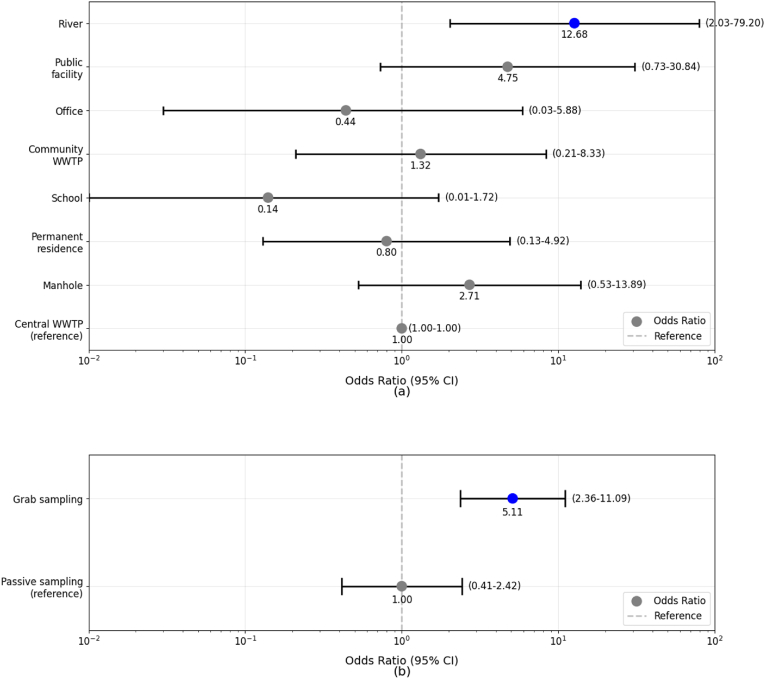
Association between the proportion of positive samples and each sampling location type (a) and sample method (b). Nodes refer to odds ratios, with blue colour showing *P* < 0.05, and gray colour showing *P* ≥ 0.05. (For interpretation of the references to colour in this figure legend, the reader is referred to the Web version of this article.)

**Fig. 5:**
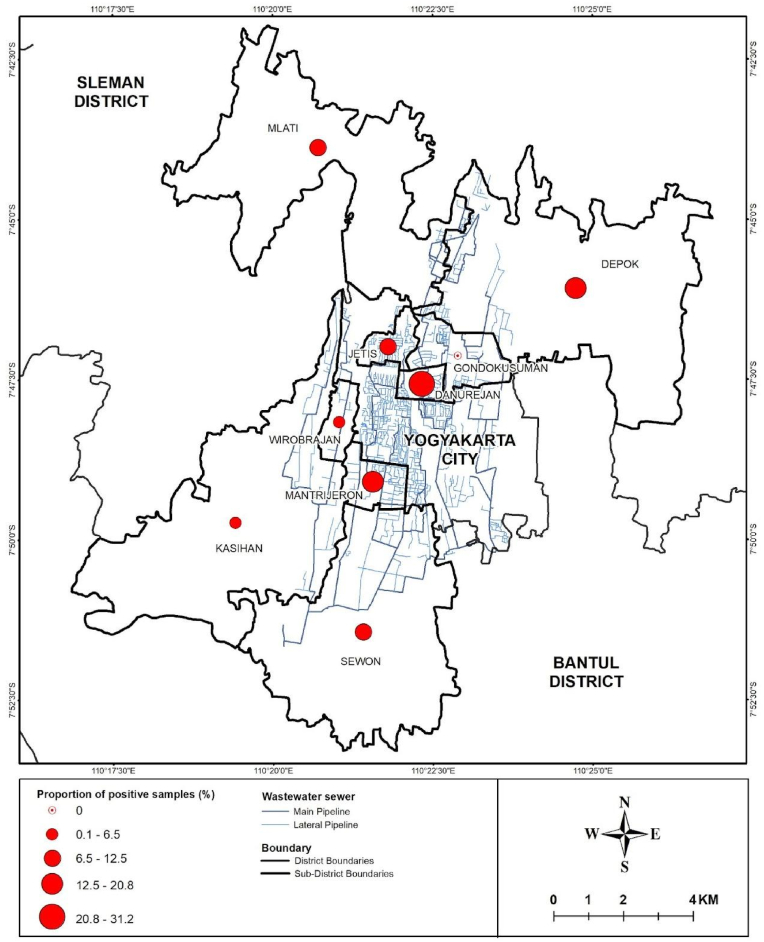
A map illustrating the population of positive samples for *S.* Typhi at each sub-district. Positivity was defined by the detection of three target genes (*ttr*, *tviB*, and *staG*), and the prevalence of positive samples was calculated by dividing the total number of positive samples by the total number of collected samples in each sub-district. Larger circle sizes indicated higher proportions of positive samples relative to the population sizes. The maps were obtained from Balai PIALAM Sewon Bantul Wastewater Treatment Plant (WWTP) and generated using ArcGIS Pro 3.4.

Samples from formal wastewater management systems (manholes, WWTPs) were also often positive (manholes 16 %, 21/120; central WWTP 8 %, 2/24); community WWTPs (10 %, 5/48). Compared to the central WWTP, the river sample had a higher positivity proportion (OR 12.68, 95 %CI 2.03–79.20, P = 0.007), after considering the week of sampling as random effects (model τ0 week = 1.46, intraclass correlation coefficient [ICC] = 0.31, Fig. 4). Samples collected from septic systems at sites serving residential areas, offices, and schools had lower positivity rates (residential areas 7 %, 5/72; offices 4 %, 1/24; schools 1 %, 1/70) (Fig. 3). Positive detection of *S.* Typhi was more concentrated in sampling locations within the urban region (Mantrijeron, Danurejan, and Jetis sub-districts) (Fig. 5).

**Fig. 6:**
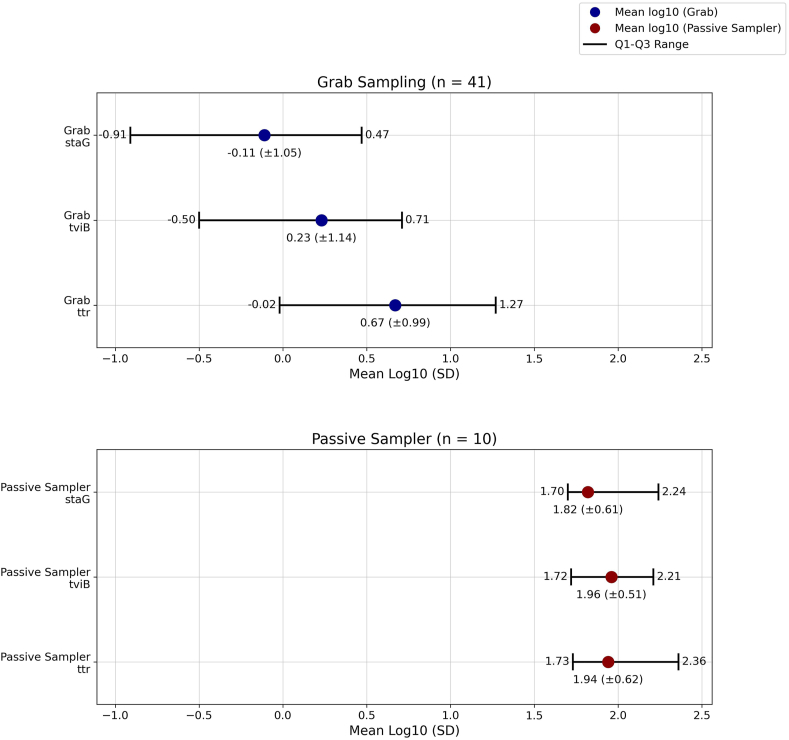
Concentration of *ttr*, *tviB*, *staG* genes among *S.* Typhi positive samples (N = 51) by sampling method (41 grab samples and 10 passive samples).

The proportion of positive detections was higher from grab samples (80.4 %, 41/51) compared to samples collected by the passive sampler (19 %, 10/51) (OR 5.11, 95 %CI 2.36–11.09, *P* < 0.001, τ_0 week_ = 1.09, ICC = 0.27) (Fig. 4). The concentration of genome copies (mean log10) in positive samples differed across the three genes (*ttr*, *tviB*, *staG*) with *ttr* having the higher concentration (copies per mL) in the positive grab samples. Due to the inherent differences in the methodology between the samples collected using the grab and passive samplers, comparison of the efficiency of the methods based on concentration (Grab: copies per mL vs. passive sampler: copies per membrane sampler) was not possible (Fig. 6). Mean concentrations (in logarithmic scale) of *ttr*, *tviB*, and *staG* in grab samples were 0.67 (SD: ±0.99), 0.23 (SD: ±1.14), and −0.11 (SD: ±1.05), respectively, while in passive samples were 1.94 (SD: ±0.62), 1.96 (SD: ±0.51), and 1.82 (SD: ±0.61). Genome concentrations in grab samples had more pronounced values smaller than one, resulting in negative values after transformation to logarithmic scale (base 10).

## 5 Discussion

This study shows that WES is feasible and consistently detects *S.* Typhi in an endemic region in Indonesia. In our study, 13 % of samples tested positive for *S.* Typhi over the study period. The highest rate of detection was from the river where 42 % samples (10/24) tested positive, with consistent detections occurring over eight months of this one-year study. A high detection rate was also observed from wastewater collected at busy markets where people frequently congregate (25 %, 6/24). Not surprisingly, positive detections were more common in urban areas compared to semi-urban and rural areas (Fig. 5). Despite a 16 % positivity rate from water collected from manholes (21/120), only 8 % of samples (2/24) collected from the central WWTP were positive, through detection using grab samples. We also observed a significantly higher probability of positive detection from the river compared to the central WWTP (OR 12.68). Whether this reflects the site-specific epidemiology of typhoid disease or a dilutional effect is unclear. However, it does suggest that WES based only on samples collected from a WWTP may significantly underestimate the prevalence of *S*. Typhi in LMICs with limited population coverage of the formal wastewater management system.

We report a higher positivity rate than observed in Malawi, (2.1 %) and India (7.5 %) (Uzzell et al., 2023), but a lower rate than reported in Bangladesh, 26.7 % (Liu et al., 2021), and Nepal (45 %) (LeBoa et al., 2023). Very few WES studies focused on *S*. Typhi from Southeast Asian countries. Within this region, Singapore has the most advanced WES system, with a broader range of pathogens tested in wastewater, such as Adenovirus, Astrovirus, non-polio Enterovirus, Hepatitis A, and Norovirus (Kilaru et al., 2023) with other countries such as Thailand, Philippines and Vietnam have focused on SARS-CoV-2 (Inson et al., 2024; Siri et al., 2024; Tangwangvivat et al., 2023). A challenge in comparing these studies is differences in methodology, including the number and position of sampling sites, duration and frequency of sampling, collection method, number of samples reported, and laboratory methods used. Current efforts are underway to standardise methods for WES in LMICs so that results can be compared across countries and regions in the future. In our study, we used both a grab method and a passive sampler method for sample collection. Grab samples were more consistently associated with positive detection for *S*. Typhi (OR 5.11 compared to the passive sampler). Grab sampling has the added advantage of being cheap and easy, but only reflects a sample collected at a single time-point. Moore swabs (gauze swabs tethered by wire or twine) have been successfully used for *S*. Typhi WES and have a higher rate of bacteria detection when compared to the membrane filter in the passive sampler (Sikorski and Levine, 2020). In this study, the passive samplers were found to be easy to deploy and retrieve, with laboratory analysis conducted using the membrane filter. However, this method was less sensitive than the grab method for the detection of *S*. Typhi. We used passive membranes rather than gauze, as in Moore swabs. Passive membranes are more sensitive for virus detection, whereas gauze-based passive samplers, like Moore swabs, may be better suited for bacterial detection (Bivins et al., 2022).

Previous studies using a single PCR target or culture-based methods have been criticised for the limited sensitivity and accuracy of detecting *S.* Typhi in wastewater (Rigby et al., 2022). This has been addressed in this study by defining the presence of *S.* Typhi by simultaneously identifying all three *S.* Typhi gene targets. The *ttr* gene is present in all strains of *S. enterica*, while *tviB* is specific to *S.* Typhi and *S.* Paratyphi *C*. The *staG* gene is highly sensitive for detecting *S.* Typhi, although this gene can also be found in non-typhoidal Salmonella serovars (Nair et al., 2019). We have used the ratio of HF183 to *ttr/tviB/staG* to distinguish human faecal contamination in wastewater samples from animal faecal contamination (Cao et al., 2018; Soller et al., 2010). HF183 is a marker for the bacteroidales group of bacteria predominantly found in the human gut, whereas *ttr/tviB/staG* can be detected in Salmonella-infected animals. However, the correlation between HF183 and *ttr/tviB/staG* may vary depending on the source of the faecal contamination, the geographic location, and other environmental factors (Ahmed et al., 2016, 2019; Fremaux et al., 2009; Haugland et al., 2010; Jardé et al., 2018; Marti et al., 2013; Nshimyimana et al., 2017; Uzzell et al., 2023).

Based on this study we found that WES for *S*. Typhi was feasible and associated with a high rate of positivity related to population density and reflected human activity around high detection sites. The estimation of the epidemiology of typhoid disease burden based on WES has been challenging due to the limitation of accurate clinical data to establish the correlation between WES detection and community typhoid burden data in most LMICs. Data on acute episodes of typhoid fever are likely underestimated in many LMICs, such as Indonesia where antibiotics are readily available without consultation with a healthcare provider. The incidence of acute typhoid fever does not capture chronic carriers who remain infected, and potentially infectious within the community. Some studies have correlated the concentration of *S*. Typhi in wastewater with clinical disease community cases, providing useful insights into the potential of WES to reflect clinical typhoid disease prevalence (Uzzell et al., 2023). However, there is limited clinical data on confirmed typhoid cases available for the Yogyakarta region. WHO recommends using typhoid conjugate vaccine TCVs to control typhoid fever in high-burden regions or regions with a high incidence of antimicrobial-resistant typhoid disease. Recommendations for national vaccination strategies (universal, risk-based, or phased) are ideally based on local surveillance data (Bentsi-Enchill and Hombach, 2019). Environmental surveillance data may be important in identifying high-burden locations to prioritise TCV campaigns (Chen et al., 2023). This is highly relevant for Indonesia, where the Indonesian Technical Advisory Group on Immunization (ITAGI) is considering the introduction of typhoid conjugate vaccines into the National Immunization Program. Additionally, there is national capacity through Bio Farma for the independent production of typhoid vaccines (International Vaccine Institute, 2023). Following vaccine introduction WES may also be a useful tool to monitor for changes in *S*. Typhi within the community.

By providing a cost-effective, non-invasive, and population-wide method of surveillance, with the adoption of emerging technologies, WES has the potential to inform targeted public health intervention and resource allocation for various infections and diseases. WES for *S.* Typhi can contribute to an improved understanding of the prevalence of typhoid disease in LMICs. Wastewater is ubiquitous and is cheap and easy to collect and is independent of more costly and complex clinical surveillance studies. This is particularly relevant in regions where typhoid fever is endemic, where infected individuals may not seek formal medical care, and where inappropriate antibiotic use is common. Specifically, WES performance was better in the context of diseases with predominantly non-specific symptoms in the early stages of the infectious period (Kilaru et al., 2023; Singh et al., 2024). WES can be used to monitor for emerging antimicrobial resistance in circulating *S*. Typhi strains.

A key limitation of this study is the lack of accurate data on the clinical prevalence of typhoid fever within the communities studied to enable the correlation of our WES data. However, this underscores the need for WES in settings where clinical identification of cases is heavily influenced by health-seeking behaviour (Carey et al., 2020) and where there are limited resources to conduct routine blood culture analysis in clinically suspected cases (Andrews et al., 2020). Our study estimated the catchment population using the local demographic data, whereas estimation using a digital elevation estimation model may provide a more precise method (Uzzell et al., 2023). Furthermore, future studies should consider the use of Moore swabs in the Indonesian setting, as this method has been proven to be more sensitive for detecting bacterial pathogens.

## 6 Conclusion

In this study, we found that WES is feasible and consistently detects *S.* Typhi in an endemic region in Indonesia. Over a one-year period, 13 % of samples were positive for *S.* Typhi*,* with the highest rate from river and urban manholes. By providing a cost-effective, non-invasive, and population-wide method of surveillance, with the adoption of emerging technologies, WES has the potential to inform targeted public health interventions, including the introduction of typhoid vaccines in Indonesia.

## CRediT authorship contribution statement

**Vicka Oktaria:** Writing – review & editing, Writing – original draft, Validation, Supervision, Resources, Methodology, Investigation, Funding acquisition, Formal analysis, Conceptualization. **Indah Kartika Murni:** Writing – review & editing, Writing – original draft, Validation, Supervision, Resources, Methodology, Investigation, Funding acquisition, Formal analysis, Conceptualization. **Amanda Handley:** Writing – review & editing, Validation, Project administration, Methodology, Investigation, Funding acquisition, Data curation, Conceptualization. **Celeste M. Donato:** Writing – review & editing, Methodology, Investigation, Conceptualization. **Titik Nuryastuti:** Writing – review & editing, Validation, Supervision, Resources, Methodology. **Endah Supriyati:** Writing – review & editing, Validation, Project administration, Methodology, Data curation. **David T. McCarthy:** Writing – review & editing, Validation, Resources, Methodology, Investigation, Conceptualization. **Emma Watts:** Writing – review & editing. **Rizka Dinari:** Writing – review & editing, Project administration, Formal analysis, Data curation. **Hendri Marinda Sari:** Writing – review & editing, Project administration, Methodology, Formal analysis, Data curation. **Jarir At Thobari:** Writing – review & editing, Supervision. **Ida Safitri Laksono:** Writing – review & editing, Supervision. **Julie E. Bines:** Writing – review & editing, Validation, Supervision, Resources, Methodology, Investigation, Funding acquisition, Conceptualization.

## Data sharing statement

The paper provides all relevant data in the supplementary material (Table A.6). Additional information on this study's protocol and analysis steps can be accessed upon request.

## Funding sources

This project was supported by the 10.13039/100000865Bill and Melinda Gates Foundation (BMGF), United States (Reference number: INV-041900). The funder of this study had no role in study design, data analysis, data interpretation, or writing of the report, and in the decision to submit the paper for publication.

## Declaration of competing interest

All authors declare no competing interests.
