# Supplementary material for: Environmental surveillance for Salmonella Typhi to detect the typhoid burden in Yogyakarta, Indonesia
Source: Int J Hyg Environ Health. 2025 May;266:114572. doi: 10.1016/j.ijheh.2025.114572 (PMC12042821; doi:10.1016/j.ijheh.2025.114572)

Supplementary Material

**Environmental Surveillance for *Salmonella* Typhi to Detect the Typhoid Burden in Yogyakarta, Indonesia**

Table of Contents

[Table A.1. Sampling location characteristics 2](#_Toc193446609)

[Table A.2. Primer and Probe Sequences 3](#_Toc193446610)

[Table A.3. Sequences Used for gBlocks gene fragments. 4](#_Toc193446611)

[Table A.4. Distribution of HF183 positive proportion and *S*. Typhi positive proportion 5](#_Toc193446612)

[Table A.5. Correlation between rainfall, *S*. Typhi positivity, and each gene (*ttr*, *tviB*, *staG*) among positive samples 6](#_Toc193446613)

[Tabel A.6. Positivity rate by sampling location and sampling batch 7](#_Toc193446614)

[Figure A.1. Interpretation of laboratory tests for typhoid 8](#_Toc193446615)

### Table A.1. Sampling location characteristics

| **No** | **Sampling Location Name** | **Sampling Source** | **Sampling Type** | **Sub-district** | **Total area (km^2^)** | **Total population** | **Total head of the family (people)** | **Population density (per km^2^)** | **Catchment population (people)** | **Estimated annual clinical cases^†^** |
| --- | --- | --- | --- | --- | --- | --- | --- | --- | --- | --- |
|  | Yogyakarta city (urban) |  |  |  |  |  |  |  |  |  |
| 1 | Manhole 1 | Manhole | Grab | Wirobrajan | 1⋅76 | 27,957 | 9,490 | 15,884 | 483* | 2 |
| 2 | Manhole 2 | Manhole | Grab | Mantrijeron | 2⋅61 | 35,469 | 12,353 | 12,773 | 979* | 6 |
| 3 | Manhole 3 | Manhole | Grab | Danurejan | 1⋅1 | 21,383 | 7,389 | 19,439 | 607* | 1^^^ |
| 4 | River 1 | River | Grab | Danurejan | 1⋅1 | 21,383 | 7,389 | 19,439 | N/A | 1^^^ |
| 5 | Traditional Market | NST water | Grab | Jetis | 1⋅7 | 27,286 | 9,493 | 16,051 | N/A | 2^^^ |
| 6 | School 1 | NST water | Passive | Jetis | 1⋅7 | 27,286 | 9,493 | 16,051 | 2,600 | 2^^^ |
| 7 | School 2 | NST water | Passive | Wirobrajan | 1⋅76 | 27,957 | 9,490 | 15,884 | 900 | 2 |
| 8 | School 3 | NST water | Passive | Gondokusuman | 3⋅99 | 43,179 | 14,836 | 10,822 | 743 | 16 |
|  | Sleman district (semi-urban) |  |  |  |  |  |  |  |  |  |
| 1 | Manhole 4 | Manhole | Grab | Mlati | 28⋅52 | 93,159 | 31,839 | 3,266 | 854* | 107^^^ |
| 2 | Residential Areas 1 | NST water | Passive | Mlati | 28⋅52 | 93,159 | 31,839 | 3,266 | 193 | 107^^^ |
| 3 | Residential Areas 2 | NST water | Passive | Mlati | 28⋅52 | 93,159 | 31,839 | 3,266 | 96 | 107^^^ |
| 4 | School 4 | NST water | Passive | Mlati | 28⋅52 | 93,159 | 31,839 | 3,266 | 183 | 107^^^ |
| 5 | Community WWTP 1 | NST water | Passive | Depok | 35⋅55 | 123,886 | 42,138 | 3,485 | 304 | 132 |
|  | Bantul district (rural) |  |  |  |  |  |  |  |  |  |
| 1 | Manhole 5 | Manhole | Grab | Sewon | 27⋅16 | 100,872 | 34,409 | 1,696 | 1,954 | 44^^^ |
| 2 | Residential Areas 3 | NST water | Grab | Sewon | 27⋅16 | 100,872 | 34,409 | 1,696 | 149 | 44^^^ |
| 3 | Office | NST water | Passive | Kasihan | 32⋅38 | 104,961 | 36,537 | 3,242 | 4,316 | 11 |
| 4 | Community WWTP 2 | WWTP (inlet) | Passive | Sewon | 27⋅16 | 100,872 | 34,409 | 1,696 | 441 | 44^^^ |
| 5 | Central WWTP | WWTP (inlet) | Grab | Sewon | 27⋅16 | 100,872 | 34,409 | 1,696 | 102,256 | 44^^^ |

*value estimates population in village level

**^†^**estimated annual clinical cases based on ICD-10 A01.0 typhoid fever, typhoid diagnosis was mainly based on signs and symptoms without microbiological confirmation through culture.

^#^Cases were not differentiated between typhoid and paratyphoid fevers

^^^Data was provided on sub-district level only so that the same sub-district will have same annual clinical case

### Table A.2. Primer and Probe Sequences

| **Primer/Probe Name** | **Sequence (5' -3')** |
| --- | --- |
| *staG_F* | CGC GAA GTC AGA GTC GAC ATA G |
| *staG_R* | AAG ACC TCA ACG CCG ATC AC |
| *staG_P* | CY5- CAT TTG TTC TGG AGC AGG CTG ACG G–BHQ3- |
| *ttr_F* | CTC ACC AGG AGA TTA CAA CAT GG |
| *ttr_R* | AGC TCA GAC CAA AAG TGA CCA TC |
| *ttr_P* | [FAM] - CA CCG ACG GCG AGA CCG ACT TT - [BHQ1] |
| *tviB_F* | TGT GGT AAA GGA ACT CGG TAA A |
| *tviB_R* | GAC TTC CGA TAC CGG GAT AAT G |
| *tviB_P* | [HEX] - TG GAT GCC GAA GAG GTA AGA CGA GA - [BHQ1] |
| HF183_F | ATC ATG AGT TCA CAT GTC CG |
| HF183_R | CTT CCT CTC AGA ACC CCT ATC C |
| HF183_P | [FAM] - CT AAT GGA ACG CAT CCC - [BHQ1] |

### Table A.3. Sequences Used for gBlocks gene fragments.

| **Gene target** | **gBlocks sequence (5' - 3')** |
| --- | --- |
| *staG* | CGGCGCGAAGTCAGAGTCGACATAGGCATAGATTTTCAGGCCATACATTAATTTGCCAAGGTTGCTATAAACATTTGTTCTGGAGCAGGCTGACGGAAATTCCGTGAACTCGCTGGTGATCGGCGTTGAGGTCTTATC |
| *ttr* | GAAACGCTGAACGGACTCACCAGGAGATTACAACATGGCTAATTTAACCCGTCGTCAGTGGCTAAAAGTCGGTCTCGCCGTCGGTGGGATGGTCACTTTTGGTCTGAGCTACCGTGATGTGGCGA |
| *tviB* | CTTGATTTGACTTCCGATACCGGGATAATGCCATACTCTCGTCTTACCTCTTCGGCATCCACCCATGGATCAAAAATATCCACTTTACAACTATATTTACCGAGTTCCTTTACCACATCAATAAT |
| HF183 | GGGATCATGAGTTCACATGTCCGCATGATTAAAGGTATTTTCCGGTAGACGATGGGGATGCGTTCCATTAGATAGTAGGCGGGGTAACGGCCCACCTAGTCAACGATGGATAGGGGTTCTGAGAGGAAGGTC |

### Table A.4. Distribution of HF183 positive proportion and *S*. Typhi positive proportion

| **HF183 positivity** | ***S*. Typhi positivity (n, %)** | | **Total (N, %)** |
| --- | --- | --- | --- |
|  | **Positive** | **Negative** |  |
| **Positive** | 51 (13,2%) | 336 (86,8%) | 387 (100%) |
| **Negative** | 0 (0%) | 19 (100%) | 19 (100%) |

### Table A.5. Correlation between rainfall, *S*. Typhi positivity, and each gene (*ttr*, *tviB*, *staG*) among positive samples

|  | **Estimate (95%CI)** | **P-value** |
| --- | --- | --- |
| All samples (N=406) |  |  |
| *S*. Typhi positivity | 0.002 (-0.253 – 0.257) | 0.989 |
| Among positive samples only (N=51) |  |  |
| Log *ttr* gene, copies per sample type | 3.794 (-1.906 – 9.494) | 0.192 |
| Log *tviB* gene, copies per sample type | 6.624 (-0.383 – 13.631) | 0.064 |
| Log *staG* gene, copies per sample type | 6.919 (-0.078 – 13.915) | 0.053 |
| Log HF183 gene, copies per sample type | 1.02 (-2.41 – 4.45) | 0.559 |

### Tabel A.6. Positivity rate by sampling location and sampling batch

| **Type** | **Sites** | **I** | | **II** | | **III** | | **IV** | | **V** | | **V** | | **VII** | | **VIII** | | **IX** | | **X** | | **XI** | | **XI** | | **XIII** | | **XIV** | | **XV** | | **XVI** | | **XVII** | | **XVIII** | | **XIX** | | **XX** | | **XXI** | | **XXII** | | **XXIII** | | **XXIV** | | **Positivity rate** | | | |  |
| --- | --- | --- | --- | --- | --- | --- | --- | --- | --- | --- | --- | --- | --- | --- | --- | --- | --- | --- | --- | --- | --- | --- | --- | --- | --- | --- | --- | --- | --- | --- | --- | --- | --- | --- | --- | --- | --- | --- | --- | --- | --- | --- | --- | --- | --- | --- | --- | --- | --- | --- | --- | --- | --- | --- |
|  |  | **10-14/10 2022** | | **24-28/102022** | | **7-11/112022** | | **21-25/112022** | | **5-9/12 2022** | | **19-23/122022** | | **2-6/01 2023** | | **16-20/012023** | | **30/01- 03/02 2023** | | **13-17/022023** | | **27/02-03/03 2023** | | **13-17/03 2023** | | **27-31/032023** | | **10-14/04 2023** | | **24-28/04 2023** | | **8-12/05 2023** | | **22-26/05 2023** | | **5-9/06 2023** | | **19-23/06 2023** | | **3-7/07 2023** | | **17-21/07 2021** | | **31/07-04/0 2023** | | **14-18/08 2023** | | **28/08-01/09 2023** | | **n** | | **%** | |  |
| Grab | Manhole 1 | *neg* | | *pos* | | *neg* | | *neg* | | *neg* | | *neg* | | *neg* | | *neg* | | *neg* | | *neg* | | *neg* | | *neg* | | *neg* | | *neg* | | *neg* | | *neg* | | *neg* | | *neg* | | *neg* | | *neg* | | *neg* | | *neg* | | *neg* | | *neg* | | 1 | | 4 | |  |
| Grab | Manhole 2 | *pos* | | *pos* | | *neg* | | *neg* | | *pos* | | *neg* | | *neg* | | *neg* | | *neg* | | *pos* | | *neg* | | *neg* | | *neg* | | *neg* | | *neg* | | *neg* | | *neg* | | *neg* | | *neg* | | *pos* | | *neg* | | *neg* | | *neg* | | *neg* | | 5 | | 21 | |  |
| Grab | Manhole 3 | *neg* | | *pos* | | *pos* | | *neg* | | *pos* | | *neg* | | *neg* | | *neg* | | *neg* | | *neg* | | *neg* | | *neg* | | *neg* | | *neg* | | *neg* | | *neg* | | *neg* | | *neg* | | *pos* | | *pos* | | *neg* | | *neg* | | *neg* | | *neg* | | 5 | | 21 | |  |
| Grab | River 1 | *neg* | | *pos* | | *neg* | | *neg* | | *pos* | | *neg* | | *neg* | | *neg* | | *pos* | | *pos* | | *neg* | | *neg* | | *neg* | | *neg* | | *pos* | | *neg* | | *pos* | | *pos* | | *pos* | | *neg* | | *pos* | | *neg* | | *pos* | | *neg* | | 10 | | 42 | |  |
| Grab | Traditional Market | *pos* | | *pos* | | *neg* | | *neg* | | *pos* | | *neg* | | *pos* | | *neg* | | *neg* | | *pos* | | *neg* | | *neg* | | *neg* | | *neg* | | *neg* | | *neg* | | *neg* | | *neg* | | *neg* | | *neg* | | *neg* | | *neg* | | *neg* | | *pos* | | 6 | | 25 | |  |
| Passive | School 1 | *neg* | | *neg* | | *neg* | | *neg* | | *neg* | | *neg* | | *neg* | | *neg* | | *neg* | | *neg* | | *neg* | | *neg* | | *neg* | | *neg* | | *neg* | | *neg* | | *neg* | | *neg* | | *neg* | | *neg* | | *neg* | | *neg* | | *neg* | | *neg* | | 0 | | 0 | |  |
| Passive | School 2^1^ | *pos* | | *neg* | | *neg* | | *neg* | | *neg* | | *neg* | | *neg* | | *NA* | | *NA* | | *NA* | | *NA* | | *NA* | | *NA* | | *NA* | | *NA* | | *NA* | | *NA* | | *NA* | | *NA* | | *NA* | | *NA* | | *NA* | | *NA* | | *NA* | | 1 | | 14 | |  |
| Passive | School 3 | *NA* | | *NA* | | *NA* | | *NA* | | *NA* | | *NA* | | *NA* | | *NA* | | *NA* | | *neg* | | *neg* | | *neg* | | *neg* | | *neg* | | *neg* | | *neg* | | *neg* | | *neg* | | *neg* | | *neg* | | *neg* | | *neg* | | *neg* | | *neg* | | 0 | | 0 | |  |
| Grab | Manhole 4 | *pos* | | *pos* | | *pos* | | *neg* | | *neg* | | *neg* | | *neg* | | *neg* | | *neg* | | *neg* | | *pos* | | *neg* | | *neg* | | *neg* | | *neg* | | *neg* | | *neg* | | *neg* | | *pos* | | *neg* | | *neg* | | *neg* | | *neg* | | *pos* | | 6 | | 25 | |  |
| Passive | Residential Areas 1 | *pos* | | *neg* | | *neg* | | *neg* | | *neg* | | *pos* | | *neg* | | *neg* | | *neg* | | *neg* | | *neg* | | *neg* | | *neg* | | *neg* | | *neg* | | *neg* | | *neg* | | *neg* | | *neg* | | *neg* | | *neg* | | *neg* | | *neg* | | *neg* | | 2 | | 8 | |  |
| Passive | Residential Areas 2 | *neg* | | *neg* | | *neg* | | *neg* | | *neg* | | *pos* | | *neg* | | *neg* | | *neg* | | *neg* | | *neg* | | *neg* | | *neg* | | *neg* | | *neg* | | *neg* | | *neg* | | *neg* | | *neg* | | *neg* | | *neg* | | *neg* | | *neg* | | *neg* | | 1 | | 4 | |  |
| Passive | School 4 | *neg* | | *neg* | | *neg* | | *neg* | | *neg* | | *neg* | | *neg* | | *neg* | | *neg* | | *neg* | | *neg* | | *neg* | | *neg* | | *neg* | | *neg* | | *neg* | | *neg* | | *neg* | | *neg* | | *neg* | | *neg* | | *neg* | | *neg* | | *neg* | | 0 | | 0 | |  |
| Passive | Community WWTP 1 | *pos* | | *pos* | | *neg* | | *neg* | | *neg* | | *pos* | | *neg* | | *neg* | | *pos* | | *neg* | | *neg* | | *neg* | | *neg* | | *neg* | | *neg* | | *neg* | | *neg* | | *neg* | | *neg* | | *neg* | | *neg* | | *neg* | | *neg* | | *neg* | | 4 | | 17 | |  |
| Grab | Manhole 5 | *pos* | | *neg* | | *neg* | | *neg* | | *pos* | | *pos* | | *neg* | | *neg* | | *neg* | | *neg* | | *neg* | | *neg* | | *neg* | | *pos* | | *neg* | | *neg* | | *neg* | | *neg* | | *neg* | | *neg* | | *neg* | | *neg* | | *neg* | | *neg* | | 4 | | 17 | |  |
| Grab | Residential Areas 3 | *neg* | | *pos* | | *neg* | | *neg* | | *neg* | | *neg* | | *pos* | | *neg* | | *neg* | | *neg* | | *neg* | | *neg* | | *neg* | | *neg* | | *neg* | | *neg* | | *neg* | | *neg* | | *neg* | | *neg* | | *neg* | | *neg* | | *neg* | | *neg* | | 2 | | 8 | |  |
| Passive | Office | *neg* | | *pos* | | *neg* | | *neg* | | *neg* | | *neg* | | *neg* | | *neg* | | *neg* | | *neg* | | *neg* | | *neg* | | *neg* | | *neg* | | *neg* | | *neg* | | *neg* | | *neg* | | *neg* | | *neg* | | *neg* | | *neg* | | *neg* | | *neg* | | 1 | | 4 | |  |
| Passive | Community WWTP 2 | *neg* | | *neg* | | *neg* | | *neg* | | *neg* | | *neg* | | *neg* | | *neg* | | *neg* | | *neg* | | *neg* | | *neg* | | *neg* | | *neg* | | *neg* | | *neg* | | *neg* | | *pos* | | *neg* | | *neg* | | *neg* | | *neg* | | *neg* | | *neg* | | 1 | | 4 | |  |
| Grab | Central WWTP | *neg* | | *neg* | | *neg* | | *neg* | | *neg* | | *pos* | | *neg* | | *neg* | | *neg* | | *neg* | | *neg* | | *neg* | | *neg* | | *neg* | | *neg* | | *neg* | | *neg* | | *neg* | | *neg* | | *neg* | | *neg* | | *neg* | | *neg* | | *pos* | | 2 | | 8 | |  |
| Weekly Positive (n) | | | 7 | | 9 | | 2 | | 0 | | 5 | | 5 | | 2 | | 0 | | 2 | | 3 | | 1 | | 0 | | 0 | | 1 | | 1 | | 0 | | 1 | | 2 | | 3 | | 2 | | 1 | | 0 | | 1 | | 3 | | 51 | |  | |
| Weekly Positivity Rate (%) | | | 41 | | 53 | | 12 | | 0 | | 29 | | 29 | | 12 | | 0 | | 12 | | 18 | | 6 | | 0 | | 0 | | 6 | | 6 | | 0 | | 6 | | 12 | | 18 | | 12 | | 6 | | 0 | | 6 | | 18 | |  | |  | |
| Monthly positivity rate (%) | | | 47 | | | | 6 | | | | 29 | | | | 6 | | | | 15 | | | | 2 | | | | | | 6 | | | | 4 | | | | 15 | | | | 9 | | | | 8 | | | | | |  | |  | |
| Positivity rate all batches | | | 13% | | | | | | | | | | | | | | | | | | | | | | | | | | | | | | | | | | | | | | | | | | | | | | | | | | | |

### Figure A.1. Interpretation of laboratory tests for typhoid


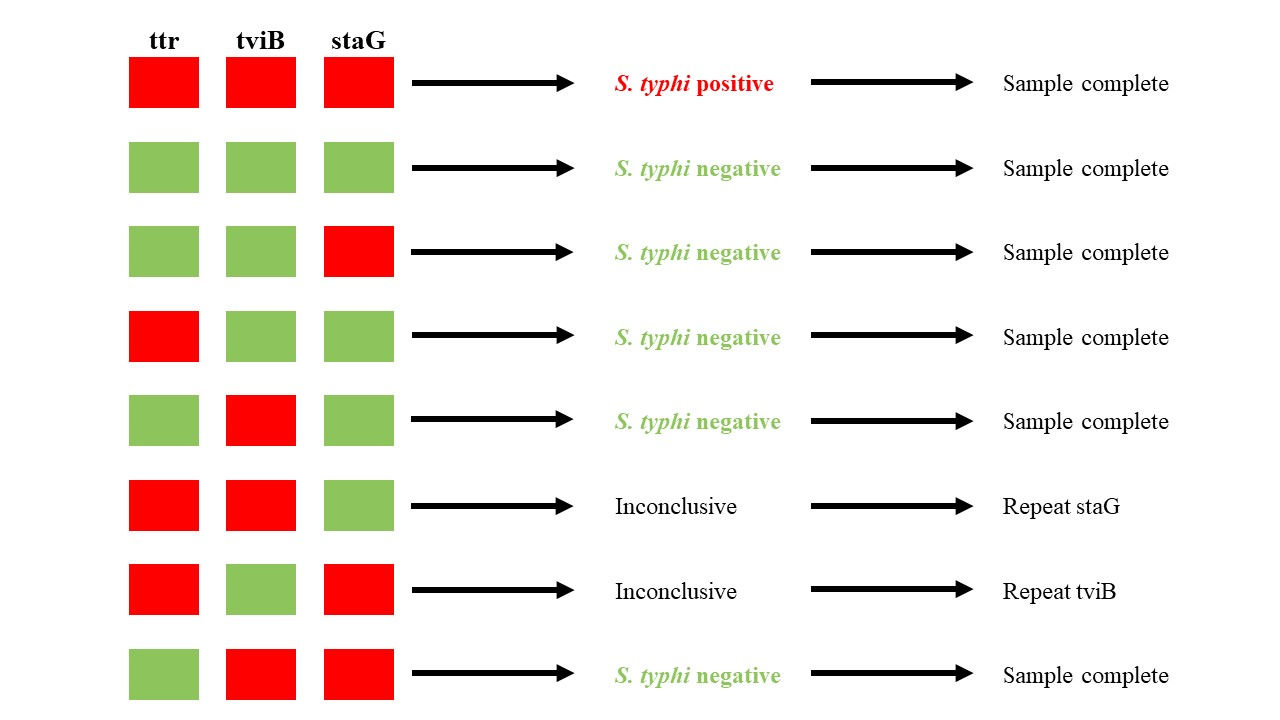

Supplement: Multimedia component 1 [file mmc1.docx]
